# Fructooligosaccharide (FOS) and Galactooligosaccharide (GOS) Increase *Bifidobacterium* but Reduce Butyrate Producing Bacteria with Adverse Glycemic Metabolism in healthy young population

**DOI:** 10.1038/s41598-017-10722-2

**Published:** 2017-09-18

**Authors:** Feitong Liu, Pan Li, Muxuan Chen, Yuemei Luo, M Prabhakar, Huimin Zheng, Yan He, Qi Qi, Haoyu Long, Yi Zhang, Huafang Sheng, Hongwei Zhou

**Affiliations:** 1 State Key Laboratory of Organ Failure Research, Division of Laboratory Medicine, Zhujiang Hospital, Southern Medical University, Guangzhou, 510282 China; 2 Department of Environmental Health, School of Public Health, Southern Medical University, Guangzhou, China

## Abstract

The gut microbiota has been implicated in glucose intolerance and its progression towards type-2 diabetes mellitus (T2DM). Relevant randomized clinical trial with prebiotic intervention was inadequate. We sought to evaluate the impact of fructooligosaccharides (FOS) and galactooligosaccharides (GOS) on glycemia during oral glucose tolerance test (OGTT) and intestinal microbiota. A randomized double-blind cross-over study was performed with 35 adults treated with FOS and GOS for 14 days (16 g/day). Faeces sampling, OGTT and anthropometric parameters were performed. Short-term intake of high-dose prebiotics had adverse effect on glucose metabolism, as in FOS intervention demonstrated by OGTT (P < 0.001), and in GOS intervention demonstrated by fasting glucose (P < 0.05). A significant increase in the relative abundance of *Bifidobacterium* was observed both in FOS and GOS group, while the butyrate-producing bacteria like *Phascolarctobacterium* in FOS group and *Ruminococcus* in GOS group were decreased. A random forest model using the initial microbiota was developed to predict OGTT levels after prebiotic intervention with relative success (R = 0.726). Our study alerted even though FOS and GOS increased *Bifidobacterium*, they might have adverse effect on glucose metabolism by reducing butyrate-producing microbes. Individualized prebiotics intervention based on gut microbiome needs to be evaluated in future.

## Introduction

The gut microbiota interacts with host and impacts on host physiology and metabolism^1^. Increasing data demonstrates that the gut microbiota played a critical role in the development of obesity^2,3^, insulin resistance^4^ and type-2 diabetes (T2DM)^5–7^. Meanwhile, blood glucose levels are rapidly increasing in the population as evident by the sharp incline in the prevalence of prediabetes^8^. Prediabetes, characterized by chronically impaired blood glucose response, is a significant risk factor for T2DM with up to 70% of prediabetes eventually developing the disease^9^. Thus, approaches that are more generously applicable to modulate gut microbiota and glucose metabolism have been widely developed^10^.

Prebiotics are non-digestible carbohydrates that beneficially affect host health by selectively stimulating the growth and/or activity of a limited number of bacteria^11,12^. Although the benefit of prebiotics have been linked to a concomitant effect of *Bifidobacteria*, no consistent conclusion has been established between prebiotics and their glucose metabolic effect. An intervention study with fructooligosaccharides (FOS) in obese women resulted in the increase of *Bifidobacterium* and *Faecalibacterium prausnitzii* but without obvious effect on glucose metabolism^13^. Moreover, our recent meta-analysis indicated that the benefit of inulin-type fructans (ITF) for reducing fasting glucose was only demonstrated in T2DM^14^. On the other hand, most of relevant prebiotics studies were based on the traditional cultivation techniques, which only focus on a limited number of species^11^.Whereas, with the development of next-generation sequencing, the understanding of gut microbiome has entered a new era and the prebiotics showed much more sophisticated effect on the whole microbiome community. For instance, an obese mice model study revealed that FOS administration increased *Bacteroidetes* and decreased *Firmicutes*, and changed more than 100 taxa of bacteria^15^. Furthermore, some researches using high-throughput sequencing have demonstrated that gut microbiota could be used to identify those subjects who would benefit from specific diet intervention^16,17^. Using personal and microbiome features enables glucose response prediction to be accurate^18^.

As typical prebiotics, FOS and galactooligosaccharides (GOS) have been widely used to stimulate the growth of *Bifidobacteria*, and in some cases *Lactobacilli* ^11^. However, to the best of our knowledge, there has been no report on the effect of FOS and GOS on human gut microbiome using the whole community profiling techniques. Thus, the aim of our exploratory study was to assess the impact of two different prebiotics FOS and GOS on glucose metabolism and gut microbiome in healthy subjects, to highlight the contribution of gut microbial changes in modulating host glucose metabolism by nutrition intervention.

## Results

### Subjects characteristics

**Figure 1:**
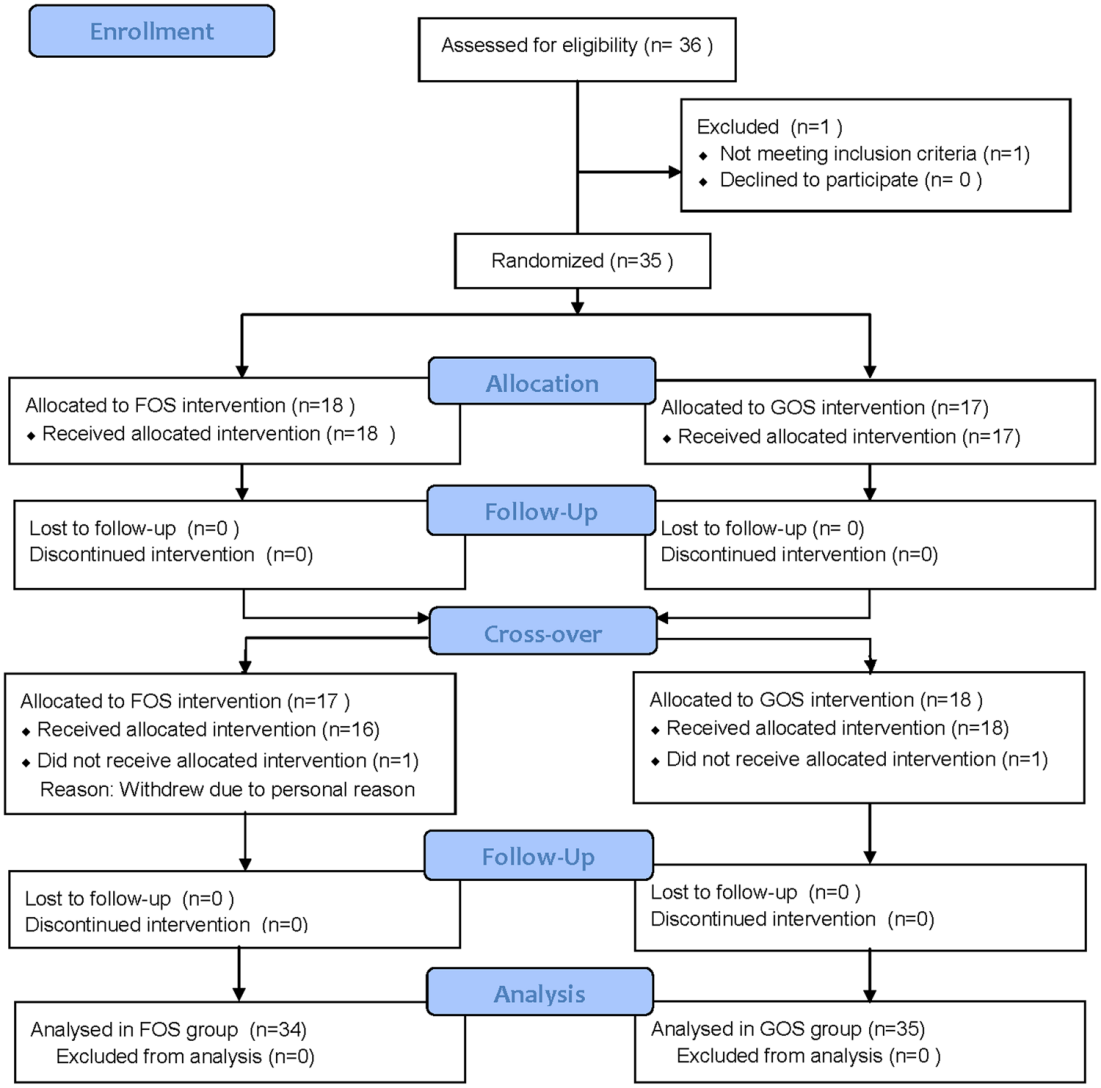
Flow chart of subject recruitment, allocation and analysis.

A total of 35 (10 males, 25 females) subjects completed the GOS intervention, 34 subjects completed FOS intervention for one women dropped out in FOS period (Fig. 1). Anthropometric and physiological data for the volunteers at the start of prebiotic intervention were shown in Table 1. There were no difference between FOS and GOS groups at the beginning of the study. Compliance and Minor side effect were reported at the end of each intervention according to the questionnaire of gastrointestinal symptoms in Supplementary Table S1. These symptoms disappeared within a few days in most participants.

**Table 1:** Anthropometric and physiological data for the participants at the start and the end of the prebiotic intervention^1^.

Characteristics	FOS group		GOS group	
	Day0	Day14	Day0	Day14
Gender	10 M 24 F	—	10 M 25 F	—
Age, y	21.9 ± 2.8	—	22.1 ± 2.7	—
Weight, kg	61.8 ± 10.4	61.5 ± 10.8	61.2 ± 10.9	61.7 ± 10.7
BMI, kg/m^2^	23.1 ± 3.3	23.0 ± 3.4	23.1 ± 3.3	23.2 ± 3.3
Fat mass, %	26.2 ± 4.6	26.2 ± 4.6	26.2 ± 5.0	26.5 ± 5.0
BMR, cal	1353 ± 213	1353 ± 223	1353 ± 219	1356 ± 219
Visceral Fat Area	4.6 ± 3.1	4.7 ± 3.1	4.7 ± 3.1	4.8 ± 3.1
Skeletal Muscle, %	28.8 ± 3.2	28.6 ± 3.3	22.1 ± 5.5	22.2 ± 5.6
Fating glucose, mmol/L	4.8 ± 0.3	4.8 ± 0.3	4.7 ± 0.5	4.9 ± 0.5*
30 min glucose level (OGTT)	8.0 ± 1.4	8.5 ± 1.2**	8.1 ± 1.1	8.5 ± 0.5
60 min glucose level (OGTT)	6.9 ± 1.4	7.9 ± 1.9**	7.4 ± 1.8	7.7 ± 1.6
90 min glucose level (OGTT)	6.1 ± 0.9	6.9 ± 1.4**	6.5 ± 1.3	6.5 ± 0.9
120 min glucose level (OGTT)	5.6 ± 0.7	6.3 ± 1.2**	6.2 ± 1.1	6.2 ± 0.7
OGTT, mmol/L	13.1 ± 1.7	14.4 ± 2.1**	13.6 ± 1.9	14.1 ± 1.9
Calorie intake (kcal)	2263 ± 176	2266 ± 192	2266 ± 191	2273 ± 190

^1^Values are mean ± SD. M, male; F, female; BMR: basic metabolism rate. *Significantly from baseline, *P* < 0.05 (Paired-Samples T Test). **Significantly from baseline, *P* < 0.001 (Paired-Samples T Test).

### Changes of anthropometric and glucose metabolism after FOS and GOS intervention

**Figure 2:**
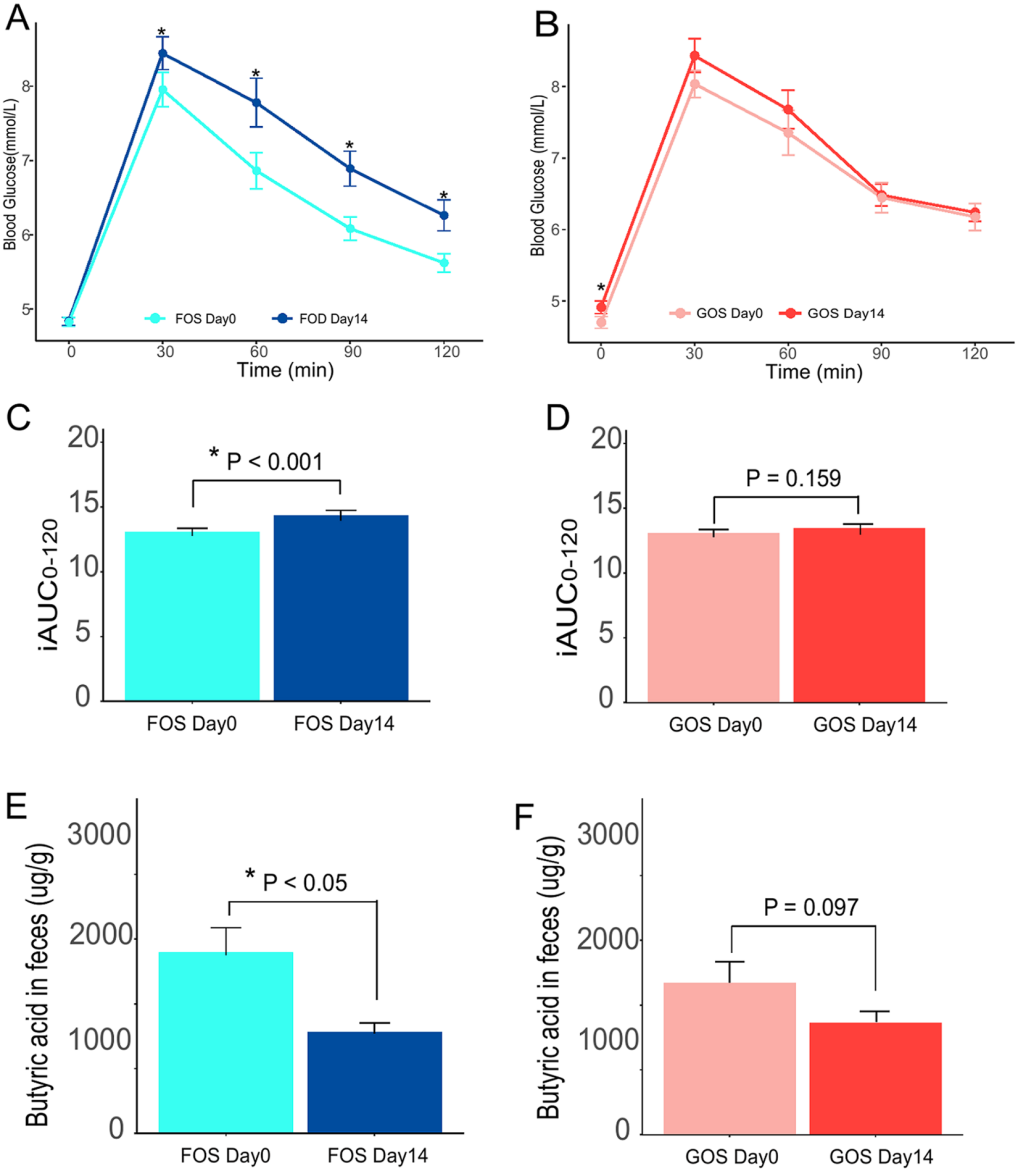
Comparison of the glucose profiles with OGTT of subjects in (**A**) and (**C**) FOS Day0 and FOS Day14; (**B**) and (**D**) GOS Day0 and GOS Day14. Comparison of the butyric acid in feces. (**E**) FOS Day0 and Day 14; (**F**)GOS Day0 and Day 14. Data are means ± SD (*p < 0.05; Paired t tests).

After 14-day intervention, neither FOS or GOS had significant impact on body weight, Body Mass Index (BMI), Body Fat, Basal Metabolic Rate (BMR), Visceral Fat Index, Skeletal Muscle and calorie intake. Similarly, the changes of blood glucose are still within the normal range (Table 1). Whereas, the glucose response significantly increased in 30 min, 60 min, 90 min, 120 min after ingesting 75 g glucose in FOS group (P < 0.001, Fig. 2A). In terms of OGTT, the increased area under blood glucose concentration curve was also identified in FOS intervention (P < 0.001, Fig. 2C), not in GOS intervention (P = 0.159, Fig. 2D). Moreover, fasting glucose was slightly increased with statistical significance after 14 days of GOS intervention (P < 0.05, Fig. 2B).

### Changes of short-chain fatty acids (SCFAs) after FOS and GOS intervention

The fecal concentration of SCFAs, including acetic acid, propionic acid, butyric acid, isobutyric acid, valeric acid and isovaleric acid, were determined by gas chromatography mass spectrum (GC-MS). The results showed that FOS significantly reduced the fecal concentration of butyric acid (P < 0.05, Fig. 2E, Table 2). A reduced tendency of butyric acid was identified in GOS intervention (P = 0.097, Fig. 2F). In addition, the concentration of acetic acid and total SCFAs were decreased after FOS and GOS intervention, but without statistical differences.

**Table 2:** Content (ug/mg) of fecal SCFAs at Day0 and Day14 with FOS and GOS intervention^1^.

Group	FOS Day0	FOS Day14	GOS Day0	GOS Day14
Acetic acid	3279.3 ± 381.7	2493.3 ± 149.7	3162.5 ± 280.6	2477.1 ± 391.0
Propionic acid	866.0 ± 80.9	887.1 ± 77.0	900.7 ± 92.5	849.6 ± 160.7
Butyric acid	1857.6 ± 272.3	1002.1 ± 82.4*	1687.0 ± 273.4	1159.8 ± 84.8
Isobutyric acid	228.5 ± 31.4	223.4 ± 26.8	218.1 ± 30.1	235.0 ± 27.9
Valeric acid	213.0 ± 122.9	61.8 ± 42.1	109.3 ± 78.2	202.1 ± 99.6
Isovaleric acid	170.4 ± 46.7	193.3 ± 25.4	167.3 ± 46.1	197.6 ± 26.0
Total SCFAs	6614.7 ± 663.2	4861.0 ± 236.2	6244.8 ± 555.9	5152.3 ± 239.0

^1^ Values are mean ± SD. *Significantly from baseline, *P* < 0.05 (Paired-Samples T Test).

### Composition of gut microbiota was profoundly altered after FOS and GOS intervention

**Figure 3:**
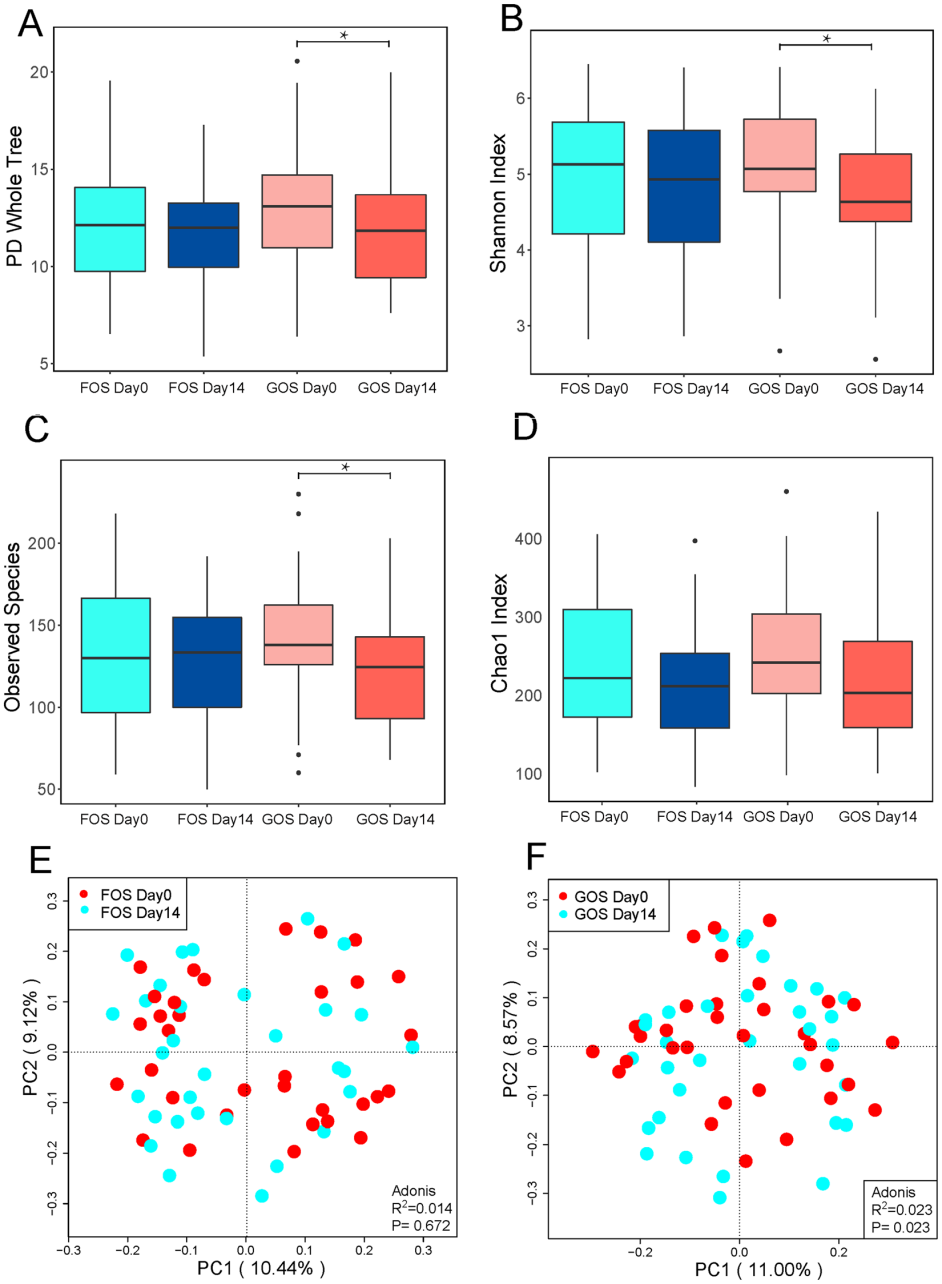
(**A**–**D**) Comparison of α-diversity between the gut microbiota of FOS Day0 and FOS Day14, GOS Day0 and GOS Day14. Four indices were used to represent the α-diversity which is (**A**) Chao1, (**B**) observed species, (**C**) Shannon index, and (**D**) PD whole tree. PD indicates phylogenetic diversity. Data are mean ± 95% CI. (*P < 0.05; Paired t tests); (**E**,**F**) Principal coordinate analysis based on the unweighted UniFrac distances. (**E**) FOS intervention, (**F**) GOS intervention. The red dots represent samples (intestinal microbiota) of pre-intervention, and the blue dots represent samples of post-intervention.

In terms of α-diversity, including species richness (represented by Chao1, observed species), phylogenetic diversity (represented by phylogenetic diversity whole tree) and richness and evenness (represented by Shannon index) of the microbial community, showed that after GOS intervention, the α-diversity was significantly lower than that before intervention. Phylogenetic diversity whole tree, Shannon index and observed species all reached statistical significance using the Wilcoxon rank sun test within GOS group (P < 0.05; Fig. 3A–D). In addition, the PCoA (a dimensionality reduction method illustrating the relationship between samples based on distance matrix) with unweighted unifrac distance indicated there was trend of separation of GOS intervention by gut microbiota (Fig. 3F, P < 0.05). PCoA visualizes the unsupervised grouping pattern of a complex data set like microbiome, and clear separation in PCoA by coloring samples from metadata indicates that the chosen information is related to microbiome. Analyses suggested that the GOS intervention was related to a significantly modification of gut microbiome, Whereas FOS intervention had no effect on α-diversity and no separation in PCoA (Fig. 3E). Moreover, after a 28-day washout period, the gut microbiota recovered to its pre-intervention state (Fig. S1).

**Figure 4:**
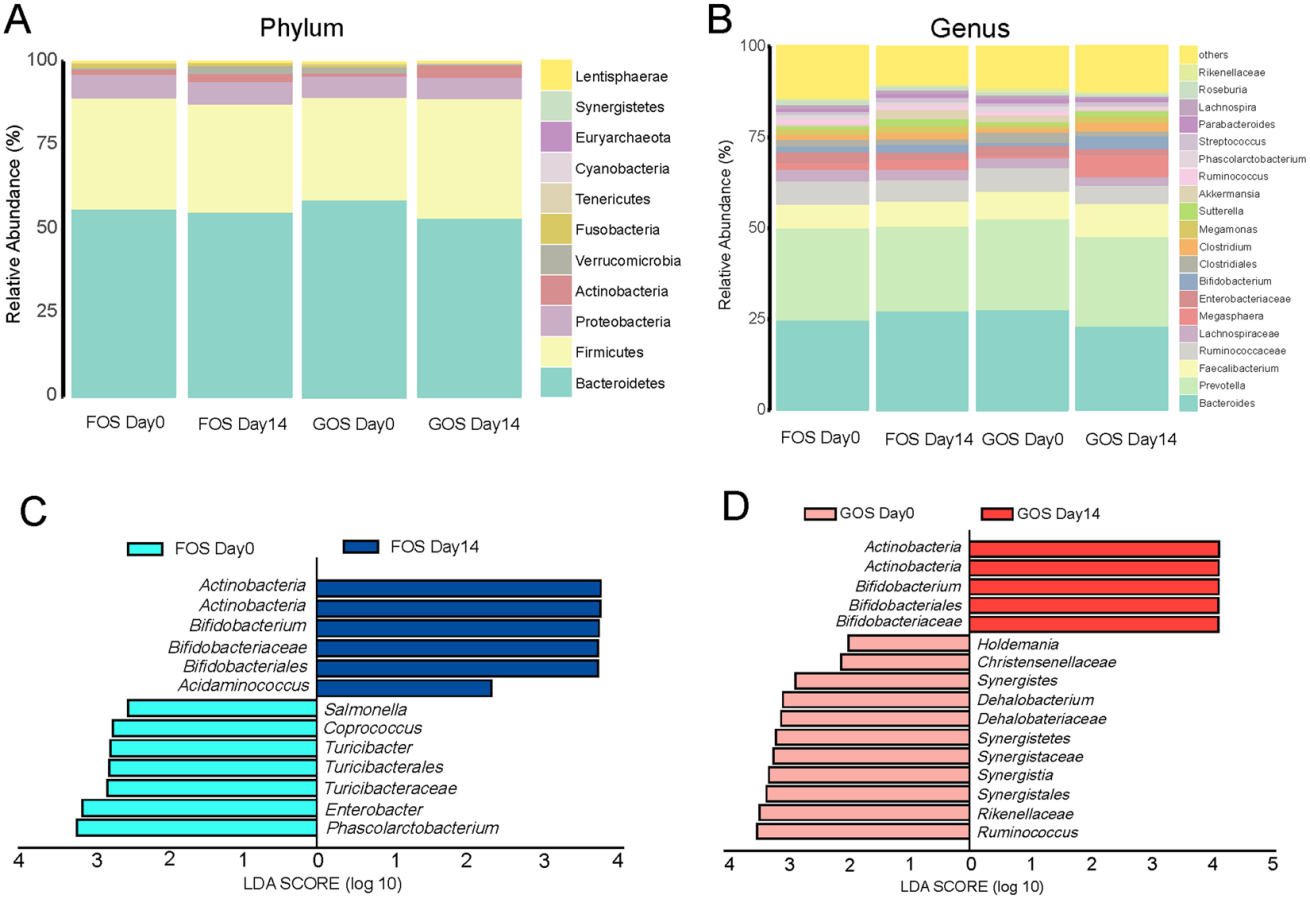
Taxonomic summary of the gut microbiota of FOS Day0 and FOS Day14, GOS Day0 and GOS Day14 at (**A**) phylum level and (**B**) genus level. Significantly discriminative taxa between (**C**) FOS Day0 and FOS Day14, (**D**) GOS Day0 and GOS Day14 determined using linear discriminant analysis effect size (LDA effect size). The red bar chart represents the bacteria that was more abundant in patients’ fecal samples, and the blue bar chart represents the controls.

Most of the gut bacteria detected in FOS and GOS group fall into 3 phyla: *Bacteroidetes, Firmicutes, and Proteobacteria* (Fig. 4A). The genus-level microbial characterization was more complex, 20 genera (mainly *Bacteroides, Prevotella, Faecalibacterium, Megasphaera and Bifidobacterium*) constituted up to 80% of gut microbiota (Fig. 4B). LEfSe analysis showed a clear difference after FOS intervention, with increased level of *Bifidobacterium* and reduced abundance of *Phascolarctobacterium, Enterobacter, Turicibacter, Coprococcus* and *Salmonella* (Fig. 4C). Similarly, the level of *Bifidobacterium* was increased and the level of *Ruminococcus, Dehalobacterium, Synergistes* and *Holdemania* was decreased after GOS intervention (Fig. 4D).

### High interpersonal variability in FOS and GOS intervention and the prediction of personalized glucose metabolism responses

**Figure 5:**
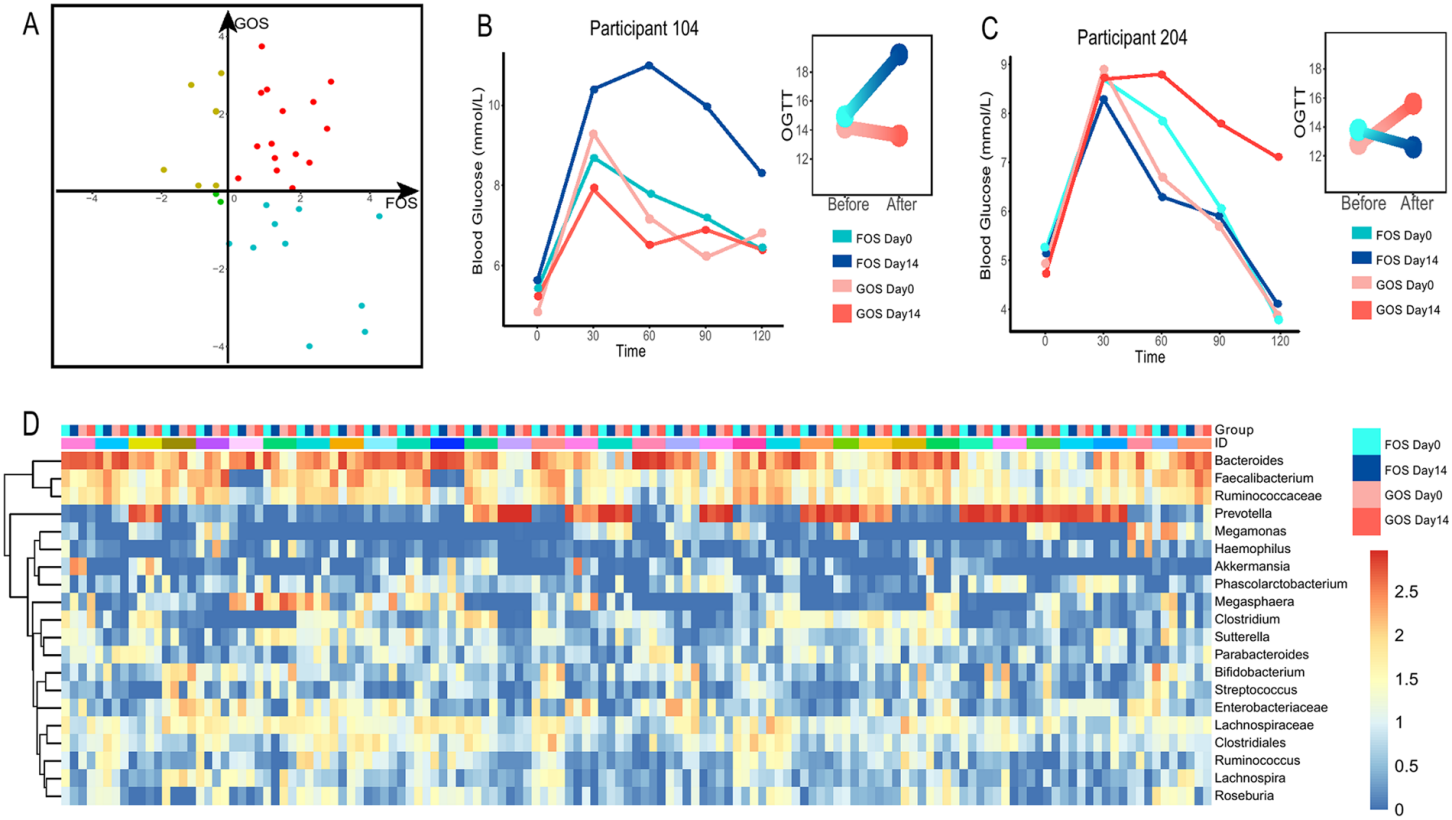
Variability of OGTT in two prebiotics intervention (**A**). Red dots represent OGTT elevated after both prebiotics. Green dots represent OGTT reduced after both prebiotics. Yellow dots represent OGTT reduced after FOS intervention, but elevated after GOS intervention; Blue dots represent OGTT reduced after GOS intervention, but elevated after FOS intervention; Glucose profiles changing with prebiotics intervention in Participants 104 and (**B**) Participants 204; (**C**) System clustering of microbiota composition at genus level. The names of several of the most abundant genera that included shown in the heatmap are listed on the right of the figure. The groups are listed at the top of the heatmap. FOS Day0, FOS Day14, GOS Day0 and GOS Day14 represent different prebiotic period. Different color represents samples from different subjects. And the color bar at the right of the heatmap shows the relative abundance of the sequences in each fecal sample.

When comparing the results of OGTT, we found high interpersonal variability (Fig. 5A). The OGTT of nine subjects was elevated after FOS, but reduced after GOS (Blue dots in Fig. 5A). Whereas, six subjects had the opposite situation. Their OGTT was reduced after FOS, but elevated after GOS (Yellow dots in Fig. 5A); For example, the glucose response of NO.104 became worse after FOS intervention but improved after GOS intervention (Fig. 5B). Whereas, in NO.204, the glucose response was improved by FOS intervention, but deteriorated by GOS intervention (Fig. 5C). In terms of gut microbiota, the increase of *Bifidobacterium* was detected in all participants except NO.118, NO.204 and NO.216. Nonetheless, other bacteria varied highly with different prebiotic intervention. Highly abundant gut microbiota and their temporal dynamics in each subject during different prebiotic intervention were demonstrated in Fig. 5D. The system clustering was formed to obtain a visual representation of the overall dynamic and each participant exhibited different microbial community.

Since significant individual differences were detected, we further used machine learning with a random forest model to test whether metadata, such as BMI, the type of prebiotics and the initial OGTT, in addition to fecal microbial taxa could predict the OGTT outcomes following the prebiotic intervention. Firstly, we developed a random forest model with the whole 16 S microbiome data, as the OGTT of each participant were predicted by tenfold cross-validation approach. By using the Recursive Feature Elimination algorithm, 40 OTUs were selected to build the optimal model. The OTUs in the model belonged to four phylum, *Bacteroidetes* (18/40), *Firmicutes* (20/40), *Actinobacteria* (1/40) and *Proteobacteria* (1/40).

**Figure 6:**
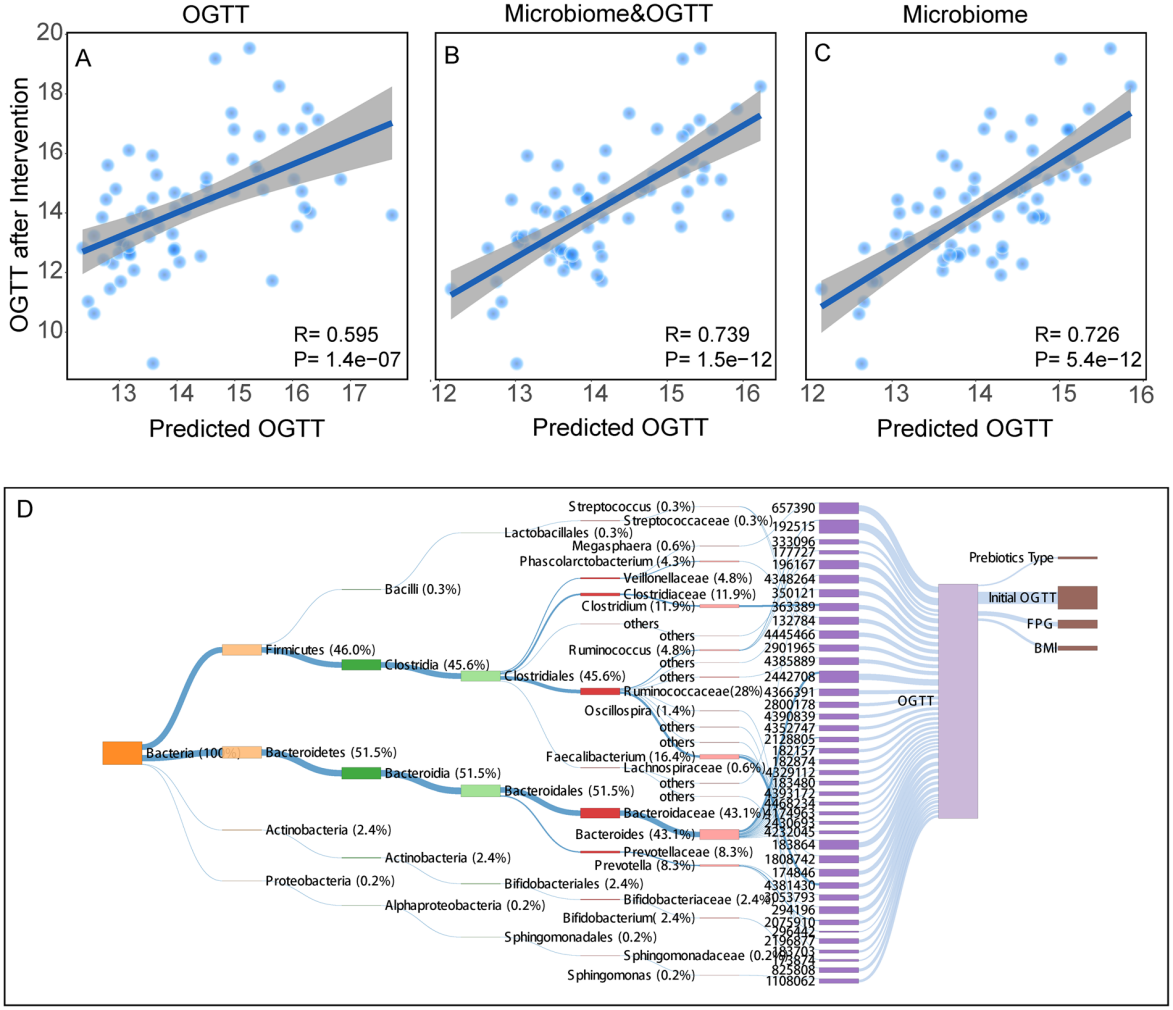
Accurate predictions of personalized OGTT responses after prebiotics intervention. (**A**–**C**) OGTT predictions. Dots represent predicted (x axis) and measured OGTT (y axis) after prebiotic intervention, for a model based: (**A**) only on the initial OGTT (**B**) on the initial OGTT and microbiome; (**C**) only on the microbiome. (**D**) Unique bacterial taxa and host factor identified in OGTT with the model. The box size to the prediction OGTT indicate the importance of the factor attributed to the OGTT outcomes. The names of the important bacterial taxa are listed on the left of the figure; the important host factors are listed on the right of the figure.

And then we only used physiological data, including the initial OGTT, BMI, body fat, BMR, visceral fat index, skeletal muscle, to build a baseline model to predict the OGTT outcomes after prebiotic intervention. The correlation coefficient between the measured OGTT values and the predicted OGTT values was statistically significant (R = 0.595, P < 10^−5^, Fig. 6A). In addition, the features that integrate the above metadata and the 40 selected OTUs predicts the following OGTT after intervention had a significantly higher correlation coefficient (R = 0.739, P < 10^−10^, Fig. 6B). The increased correlation for the model added with microbiota indicated that the initial fecal bacterial community correlated better with the OGTT outcomes than these known risk factors of glucose intolerance^19^. Of interest, only using the microbial taxa (40 selected OTUs), the correlation coefficient between the measured OGTT values and the predicted OGTT values was still higher than that only using physiological data (R = 0.726, P < 10^−10^, Fig. 6C).

According to the model results, further exploration indicated that the initial OGTT, fasting glucose, and BMI were the host factors correlated with the predicted OGTT (Fig. 6D). We further picked the bacterial lineages of importance for modeling and observed that the initial microbiome had a better explanation for the prediction model than the physiological parameters. The results demonstrated that *Bacteroides, Faecalibacterium, Clostridium, Prevotella, Ruminococcus, Veilllonellaceae, Phascolarctobacterium* and *Bifidobacterium* were all correlated with the OGTT outcomes. It was noticed that the genera *Bacteroides* contributed most to the prediction model. Meanwhile, some universal butyrate-producing bacteria, such as *Faecalibacterium, Ruminococcus* and *Phascolarctobacterium*, also correlated with OGTT outcomes in the prediction model. Whereas, *Clostridium*, which was identified as highly discriminant for T2DM, were also correlated with the OGTT outcomes. Overall, these findings demonstrated that the initial gut microbiome had the potential to predict OGTT outcomes after different types of prebiotic intervention.

## Discussion

The gut microbiota is considered as an organ composed of a large diversity of bacterial cells that can perform different functions. Nutritional approaches are considered potential tools to modulate the gut microbiota with a concomitant impact on health^20,21^. Thus, the concept of prebiotics has been increasingly proposed as modulators of microbial ecology and physiology in humans. Especially with the popularity of high throughput sequencing techniques, the effects of prebiotics have been observed throughout the whole gut microbiota community. Meanwhile, as a kind of functional food, prebiotics were increasingly paid more attention to improving glucose metabolism^22,23^. Thus, this study explored the effect of FOS and GOS on glucose metabolism and gut microbiota, furthermore, established a model using microbial data to predict glucose metabolism with relative success.

Impaired glucose response is a significant risk factor for T2DM. Thus, maintaining good blood glucose response is considered critical for preventing and controlling the metabolic disease^24^. In our study, for the deeper look at the glucose metabolism, fasting glucose and OGTT were both measured. For the first time, this study demonstrated that short-term administration with high-dose FOS and GOS had adverse effect on glucose metabolism. However, there are some differences between two prebiotics: as in FOS intervention demonstrated by OGTT (P < 0.001), and in GOS intervention demonstrated by fasting glucose. One possible explanation is that even though they all reduced the butyric acid, the reduce degree is differed. In FOS group, the fecal concentration of butyric acid was significantly decreased by 46.1%, whereas the reduce of butyric acid in GOS group was only a trend with 31.2%. SCFAs, especially butyric acid produced in the distal gut by bacterial fermentation that might improve T2DM features^25^. Their main beneficial activities were identified in the decrease of serum levels of glucose, insulin resistance as well as inflammation and increase in protective Glucagon-like peptide (GLP-1)^25^. Meanwhile, GLP-1 is an incretin hormone that participates to glucose homeostasis, mainly by lowering plasma glucose level, improving insulin secretion and resistance^26^.

On the other hand, the alteration of glucose observed in our study is slightly different from other literature. Previous studies focused on glucose metabolism reported daily intake of FOS or inulin had no effect on fasting glucose^13,27–31^, and OGTT was tested negative^13,32^. Through comparing the methodology of related trials, the difference may be attributed to the types and dose of prebiotics. For example, the FOS used in our trial was produced via the hydrolyzation of sucrose, with sucrose as the raw material. In the other trials, the FOS was a natural product extract^31^ or was mixed with inulin^13,27^. Moreover, the prebiotics dose used in our trial was 16 g per day (the maximum dose according to national standards). All these factors may have impact on the results, especially on the most sensitive indicator like blood glucose. Thus, further researches focused on the different prebiotics source and dosage relationship are needed in the future.

In our study, daily prebiotics supplementation had a selective modulation of gut microbiota. The worsen α-diversity was observed with the adverse fasting glucose after GOS intervention. Consistent with this, Gordon *et al*. and Larsen *et al*. reported that α-diversity was decreased in obese^33^ and T2DM population^34^. Moreover, our recent study suggested that individuals with metabolic risk factor were characterized with lower gut microbiome richness^35^. At phylum level, *Actinobacteria* was increased in both prebiotics group. The increased level of *Actinobacteria* following prebiotic treatment was due to the increase in *Bifidobacterium*. A large number of studies in adult participants consistently showed significant bifidogenic changes in the gut microbiota after consumption of prebiotics^36–38^. Meanwhile, Meyer *et al*. noted that *Bifidobacterium* do not contain any known pathogens, and they are primarily carbohydrate fermenting bacteria, unlike other groups such as *Bacteroides* and *Clostridia* that are also proteolytic and amino acid fermentation. Thus, *Bifidobacterium* can fully ferment non-digestible carbohydrate into lactic acid and acetic acid, which can be utilized by host as energy sources^36^.

By contrast, at the genus level, the prebiotic intervention decreased butyrate-producing bacteria, such as *Ruminococcus, Phascolarctobacterium, Coprococcus* and *Oscillospira*, all these bacteria have been reported to be able to produce butyrate with anti-inflammation effect^39,40^. In consistent with the butyrate-producing bacteria, the concentration of butyric acid was also decreased after prebiotics intervention. Some researches regarding to T2DM indicated that the loss of these butyrate-producing bacteria was associated with the impaired glucose control^4,5^, which was similar to our observation of the deteriorated glucose response following the decreased butyrate-producing bacteria with prebiotic intervention. Moreover, the prebiotic intervention also decreased some opportunistic pathogens, such as *Enterobacter* and *Salmonella*, which have previously been reported to cause or underlie human infections such as bacteraemia and intra-abdominal infections^41^. With all the results together, we inferred that high-dose prebiotics intervention mainly promoted the proliferation of *Bifidobacterium* with producing much lactic acid, inhibiting the growth of opportunistic pathogens, also hindering the growth of butyrate-producing bacteria and SCFA production, which may be related with deteriorated glucose metabolism.

High interpersonal variability in the oral glucose tolerance response to identical prebiotic intervention was observed in this study. Similarly, the gut microbiome also varied with different types of prebiotic intervention in individuals. This observation was consistent with a very recent report that variable responses of human microbiome to dietary supplementation with resistance starch (RS)^16^. Venkataraman *et al*. proposed that the heterogeneous responses in butyrate concentrations upon RS supplementation may be explained by the characteristic of gut microbiota. In our study, further analysis using machine learning indeed suggested a correlation between gut microbiota and the OGTT after prebiotics intervention. The model showed that *Faecalibacterium, Ruminococcus* and *Phascolarctobacterium* correlated well with OGTT outcomes. These bacteria are known human gut colonizers and butyrate producers^37^, and have been linked to improved insulin sensitivity and diabetes amelioration^42,43^. These results were supported by the LEfSe analysis, as demonstrated by the fact that a lower level of *Ruminococcus* was present with an impaired glucose response after FOS intervention. Similarly, a lower level of *Phascolarctobacterium* was present with an increased fasting glucose after GOS intervention. With a wider application, the glucose metabolism prediction model can help to determine whether a kind of prebiotic is appropriate for specific individual and further provide a better personalized nutrition suggestion. More researches are needed to examine the correlation and explore the underlying mechanism.

Still, the present study has its own limitations and calls for improvement in future related researches. Firstly, the duration of prebiotics intervention was relatively short and the metabolic evaluation indicators are not comprehensive; secondly, due to the constraints of the sample size, self-control and cross-over methods was adopted; at last, because the inclusion criteria were restricted within healthy subjects, the conclusion requires further investigation and to be generalized to the whole-population and specific disease.

## Material and Methods

### Ethical Issues

The study was designed according to the CONSORT 2010 (Supplementary Consort Checklist). This randomized double-blind self-controlled trial was conducted at Southern Medical University Guangzhou, China. The study protocol was reviewed and approved by the Chinese Ethics Committee of Registering Clinical Trials (No. ChiECRCT-20160021). All enrolled patients provided written, informed consent for the study. The study was performed in accordance with the principles of Declaration of Helsinki and registered at www.chictr.org.cn (number ChiCTR-IPR-16008460) on 5/11/2016. All methods for each subject were performed in accordance with the approved ethical guideline and there was no change made to this trial after the commencement of recruitment.

### Subjects

Thirty-six subjects of both genders between the age of 18–65 years were voluntarily recruited from December 2015 to May 2016. The variable chosen for the calculation of sample size was fasting glucose and the specific methods are detailed in Supplementary Methods online. Exclusion criteria were: BMI < 18 kg/m^2^, recently intake (<3 months) of antibiotics or drugs known to influence gut microbiota composition, intake of probiotics or fiber supplements, use of antidiabetic drugs or weight-loss treatment, the presence of anaemia, gastrointestinal disorders or chronic disease, pregnancy and lactation, unusual dietary habits (vegetarians and vegans).

### Trial protocol

After confirming participants were in accordance with the inclusive criteria and obtaining consent, the participants were assigned to dietitians and received guides for keeping food diary daily with a smartphone application software named “Boohee” (Shanghai Boohee Information Technology Co., Ltd). After a run-in period of one week, the participants were randomized to FOS or GOS group following a systematic allocation method. Each of the treatment performed with daily supplement of 16 g FOS (QHT-Purity95%) or GOS (QHT- Purity95%) (8 g twice a day) and lasted two weeks. Then the subjects went through a four-week washout period to avoid the carry-over effects and then they were crossed over to the other treatment. Prebiotics products were kindly provided by Quantum Hi-Tech (China) Biological company, Guangdong, China (the characteristics of prebiotics were shown in Supplementary Table S2). The subjects were recommended to take a half dose during the first two days to promote adaption to the prebiotics and minimize gastrointestinal symptoms. Both FOS and GOS were provided in identical opaque packages. The powder was suggested to add in drinks such as coffee, tea or dairy products. During the whole study, the participants were asked to maintain their lifestyle and eating habit, avoid consumption of yoghurt which contains FOS or GOS.

The participants were asked to collect fecal samples at each pre-intervention (FOS Day0, GOS Day0) and post-intervention (FOS Day14, GOS Day14). Fecal samples were frozen at −80 °C within one hour of excretion. The content of fecal SCFAs was quantified by GC-MS and the specific methods are detailed in Supplementary Methods. At the beginning and the end of each treatment, the participants were also arranged to an oral glucose tolerance test (OGTT) after an overnight fasting for 12 hours. 75 g glucose in drinking solution and measurement of glycaemia at 0 minutes, 30 minutes, 60 minutes, 90 minutes and 120 minutes after ingestion. Meanwhile, body composition was measured by a whole-body electrical resistance analyzer (Omron HBF-701, Omron health medical China, Dalian, China). Body weight, BMI, body fat (%), body metabolic rate (BMR), visceral fat area, skeletal muscle (%) were determined. Daily dietary caloric intake was assessed by dietitians. Moreover, participants were asked to fill out questionnaires about gastrointestinal symptoms, including bloating, flatulence, abdominal pain, increased frequency of defecation and farting, increased appetite or loss of appetite. The Wechat follow-up (Shenzhen Tencent Computer System Co., Ltd) was performed daily to verify compliance and record possible side effects. The final compliance was assessed by the numbers of fecal samples and counting unused sachets that participants were asked to return at the end of each intervention. All participants and researchers were blinded to the whole intervention.

The primary outcome of the study was the effect of FOS and GOS on glucose metabolism and the composition of gut microbiota. The effect of prebiotics on body composition profiling and side effects was then evaluated as the secondary outcome.

### Gut microbiota compositional analysis and establishment of the prediction model

Fecal samples were collected and stored at −80 °C until further processing. The DNA was isolated with DNA automatic extraction machine (Allsheng Auto-Pure20 Nucleic Acid Purification System, Hangzhou, China) as we previously described^44^, using the Fecal DNA nucleic acid extraction kit (Shenzhen Bioeasy Biotechnologies, Inc., China) as per the manufacturer’s instructions. The V4 variable regions of bacterial 16 S rRNA gene was amplified by polymerase chain reaction (PCR) using forward primers 514 F (GTGTGCCAGCMGCCGCGGTAA) that contained a sample-specific barcode with an Ion A adaptor (CCATCTCATCCCTGCGTGTCTCCGACTCAG), while the associated reverse primer 805 R (CCGGACTACHVGGGTWTCTAAT) contained truncated P1 adaptor (CCTCTCTATGGGCAGTCGGTGAT). The PCR cycle conditions were described previously^44^. Fecal microbiota composition was assessed using partial 16 S rRNA sequences that were determined on a 318 V2 chip using the Ion Torrent Personal Genome Machine System in Public Health School, Southern Medical University. The raw sequences were preprocessed according to the BIPES protocol^45^.

Data analysis were performed in QIIME1.8 framework as follows^46^. Samples with less than 1000 reads have been exclude from analysis. Sequences were clustered into operational taxonomic units (OTUs) using the Usearch algorithm^47^. Representative sequences for each OTU were determined based on sequences frequencies; representative sequences were aligned using PyNAST algorithms^48^. Phylogenetic relationships were determined based on representative sequence alignment using FastTree^49^. Taxonomic assignments for each representative sequence were determined; and the above information was combined to construct the BIOM file^50^. We used the command of beta_diversity_through _plots.py –i otu.biom –o output_dir for the principal coordinate analysis (PCoA). All samples were normalized for the subsequent analysis. The sequences were deposited in the European Nucleotide Archive (ENA), with accession number PRJEB15149. Metadata, OTU table have all been included as Supplementary Dataset S1 and S2.

To determine the significantly differential taxa between pre-intervention and post-intervention, we applied linear discriminate analysis size effect (LEfSe) to compare samples between two timepoints^51^. The linear discriminant analysis (LDA) threshold was set to 2. LEfSe is an algorithm for high-dimensional biomarker discovery; it determines the features most likely to explain differences between classes by coupling standard tests for statistical significance with additional tests encoding biological consistency and effect size. A LDA value will be calculated for each of the differential features detected by LEfSe, and that value represents the differences of this feature between tested groups.

Random forest regression models were built of the default set of 1000 trees, with the caret R package to predict the OGTT level after prebiotics intervention. Training was achieved through 10-fold cross validation with OTUs data as well as the blood glucose and anthropometry data. The feature selection was performed by using the recursive feature elimination algorithm of the caret R package^52^. The importance scores of features were determined based on the increase of prediction error when that feature was randomly permuted while all others were remained unchanged^53^. The correlation coefficient (Pearson) between the predicted OGTT value and the measure value were calculated with R.

### Statistical Analyses

Raw data are expressed as mean ± SD. Statistical analyses were performed using R (3.0.2). A full record of all statistical and bioinformatic analysis is included in Supplementary Method. Treatment effects of FOS and GOS were assessed by comparing the value at Day0 and Day14 for each subject using Paired-Samples T Test, as most of the parameters had an normal distribution (assessed using a Shapiro-Wilk test). The Wilcoxon rank sum test was used for the test indices not passed the the Shapiro–Wilk normality test. Because the microbiome data are multidimensional, we used the Adonis test implemented in QIIME 1.8.0. A value of P < 0.05 was considered as statistically significant in the compared groups.

## Electronic supplementary material

Supplementary information

Dataset 1

Dataset2
