# Supplementary material for: Fructooligosaccharide (FOS) and Galactooligosaccharide (GOS) Increase Bifidobacterium but Reduce Butyrate Producing Bacteria with Adverse Glycemic Metabolism in healthy young population
Source: Sci Rep. 2017 Sep 18;7:11789. doi: 10.1038/s41598-017-10722-2 (PMC5603605; doi:10.1038/s41598-017-10722-2)
Supplement: Supplementary file 1 — Supplementary information [file 41598_2017_10722_MOESM1_ESM.pdf]

Supplementary Information

Fructooligosaccharide (FOS) and Galactooligosaccharide (GOS) Increase Bifidobacterium but Reduce Butyrate Producing Bacteria with Adverse Glycemic Metabolism in healthy young population

Feitong Liu, Pan Li, Muxuan Chen, Yuemei Luo, M Prabhakar, Huimin Zheng, Yan He, Qi Qi, Haoyu Long, Yi Zhang, Huafang Sheng, Hongwei Zhou*

Supplementary Figure S1

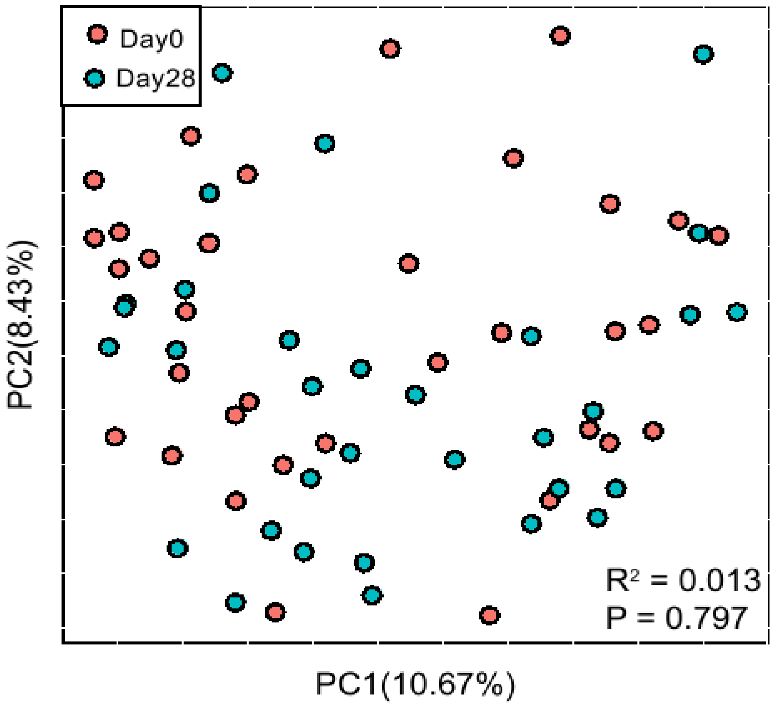

The gut microbiota recovered to its pre-intervention state after a 28-day washout period.

Supplementary Table

Table S1 Gastrointestinal symptoms of participants in two group

Compliance and Symptoms	FOS group	GOS group
Compliance	97.5%	97.6%
Bloating and flatulence	9/34	12/34
Abdominal pain	4/34	5/34
Increased frequency of defecation	7/34	10/34
Increased frequency of farting	24/34	21/34
Increased appetite	4/34	7/34
Loss of appetite	7/34	6/34

Table S2 Character of FOS and GOS used in the study

Character	FOS	GOS
Source	Sucrose	Lactose
Molecular formula	$C_6(n+1)(H_2O)(_{6+5}n)$ (2≤n≤4);	$(C_6H_{11}O_5)$ n (2≤n≤5)
Ratio % (n=2)	61.8	16.6
Ratio % (n=3)	34.0	42.4
Ratio % (n=4)	4.2	25.6
Ratio % (n=5)	--	15.4
Molecular Weight	504-828	326-815
Purity	90%	90%

Supplementary Methods

1、Estimation of sample size

The sample size was determined by fixing the probability of type I error at 0.05 (α =0.05) and that of type II error at 0.10 (β =0.1). The variable chosen for the calculation of sample size was fasting glucose; the minimum difference we wish to detect is 0.40 mmol/L with the expected standard deviation (SD) of 0.40. The sample was determined size by the formula: N=2*[(Zα ＋Zβ)* σ/d] ^2. In this case, Zα =1.96，Zβ =1.28, d=0.4, σ =0.4. Thus, the sample size was calculated, N=22. And considering the lost to follow-up, the number of volunteer in each group must not be less than 30.

2、Fecal Short-chain Fatty Acids (SCFAs) Quantification by GC-MS.

Quantification analysis of fecal SCFAs are same as the described method[1] and performed using an Agilent 7890A gas chromatography coupled with an Agilent 5975C mass spectrometric detector (Agilent Technologies, USA). For feces samples, fecal water was prepared by homogenizing feces in 0.005 M aqueous NaOH followed by centrifuging at 13,200 g at 4 °C for 20 min. The supernatant fecal water was derivatization with PrOH/Pyridine mixture solvent (3:2, v/v) and propyl chloroformate (PCF). After derivatization, the derivatives were extracted by a two-step extraction with hexane. The concentrations of the SCFAs (acetic acid, propionic acid, butyric acid, isobutyric acid and n-valeric acid) were performed with a polar DB-WAX capillary column (30 m × 0.25 mm i.d., 0.25 µm film thickness, Agilent, CA). Helium was used as a carrier gas at a constant 137 flow rate of 1 mL/min. The initial oven temperature was held at 60 °C for 5 min, ramped to 250 °C at a rate of 10 °C/min, and finally held at this temperature for 5min. The temperature of the front inlet, transfer line and electron impact (EI) ion source were set as 280, 250 and 230 °C, respectively. Data handing was performed with an Agilent's MSD ChemStation (E.02.00.493, Agilent Technologies, Inc., USA).

1 Zheng, X.; Qiu, Y.; Zhong, W.; Baxter, S.; Su, M.; Li, Q.; Xie, G.; Ore, B. M.; Qiao, S.; Spencer, M. D.; Zeisel, S. H.; Zhou, Z.; Zhao, A.; Jia, W., A targeted metabolomic protocol for short-chain fatty acids and branched-chain amino acids. Metabolomics. 2013, 9, 818-827.

1\ Total Ion Chromatogram in Standards.

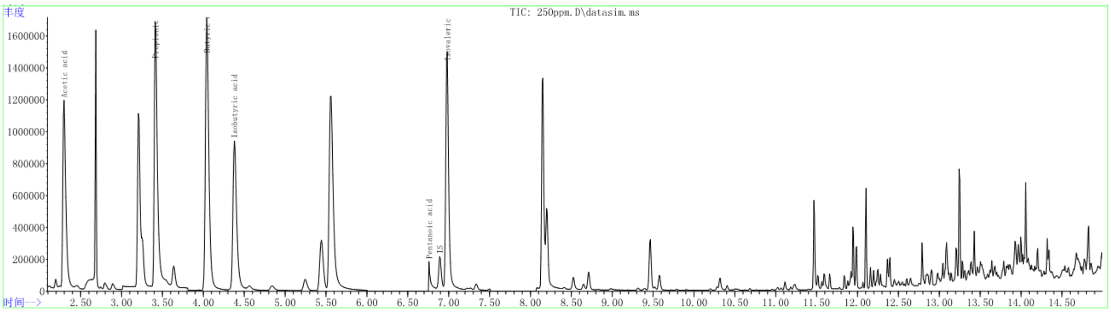

2\ Total Ion Chromatogram in Samples.

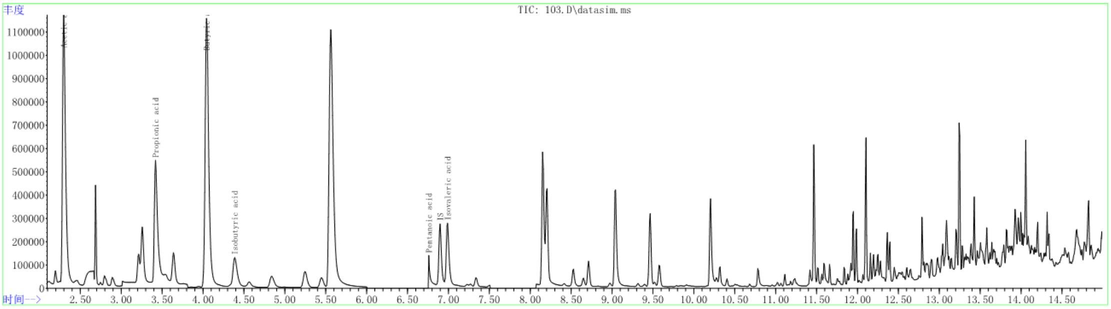

3、Statistical analysis and bioinformatic analysis

OUT location：

FnP: ~/projects/prebiotics.liufeitong/repeat.all.steps/ otu_table.prebiotics.biom

standardize the total otu count to 1007

single_rarefaction.py -i otu_table.prebiotics.biom -o otu_table.prebiotics.even1007.biom -d 1007

107BF9, 209CF5, 107DG9, 107CF5 had been dropped.

Alpha-diversity

alpha-diversity calculation

alpha_diversity.py -i otu_table.prebiotics.even1007.biom -m chao1,PD_whole_tree,shannon,observed_species -o adiv.txt -t rep_set.tre

alpha-diversity plotting and statistic

combine metadata with alpha-diversity file and then ,

Use R script: alpha-div.R

Beta-diversity

Use Qiime 1.9 to calculate beta-diversity:

beta_diversity_through_plots.py -i otu_table.prebiotics.even1007.biom -o bdiv -m metadata.txt -t rep_set.tre -p bdiv.params.txt

Split metadata&distance matrix into FOS/GOS respectively:

Delete the “#” in the first unit of metadatas. (“#SampleID” to “SampleID”)

Use R script: metadata_split.r

Add “#” to the first unit of metadatas.

Adonis

compare_categories.py --method adonis -i dm.FOS.txt -m meta.FOS.txt -c compareF9 -o adonis_F -n 999

compare_categories.py --method adonis -i dm.GOS.txt -m meta.GOS.txt -c compareG9 -o adonis_G -n 999

PCoA plotting

Delete the “#” in the first unit of metadatas. (“#SampleID” to “SampleID”)

Use R scripts: unweighted.pc.to.2D.r

TAXA

Make taxonomy file

summarize_taxa_through_plots.py -i otu_table.prebiotics.even1007.biom -o taxa -m metadata.txt -c stage

L2 level plotting

Delete the “#” units

Use taxa.L2.R to plot

L6 level: select top 20 genus and others add up to others

Delete the “#” units

Select the top 20 abundance genus and sum up other genus to “others”.

To sort the genus, the order index of “others” set to 0(min).

Use taxa.L6.R to plot

LefSe

Create taxonomy charts of all samples

summarize_taxa_through_plots.py -i otu_table.prebiotics.even1007.biom -o taxa.all -m metadata.txt

Heatmap

Make taxonomy file

summarize_taxa_through_plots.py -i otu_table.prebiotics.even1007.biom -o taxa.all -m metadata.txt

L6 level: select top 20 genus and others add up to others

Two files are needed: the taxa-abundance file and the grouping file.

1. Prepare the taxa-abundance file

Delete the “#” units and the first cell (keep the first cell blank)

Select the top 20 abundance genus

Transposition

2. Prepare the grouping file

Delete the “#” units and the first cell (keep the first cell blank)

Use R script: heatmap.R to plot

Blood Glucose Analyses

This session contains three parts:

Basic blood glucose analysis;

The point plot of the difference values between FOS/GOS intervention

The analysis of individual 104 & 204

Use the R script: BloodGlucoseAnalysis.R

Random Forest Model

RFE to select features for a better model

Create args file:

--input_otu_table otu_table.prebiotics.even1007.txt

--metadata meta.model.txt

--fields OGTT

--models rf

--add_category stage

--feature_selection

Pick OTUs as the RFE suggested

Run model with all features

Create args file:

--input_otu_table otu_table.prebiotics.even1007.txt

--metadata meta.model.txt

--fields OGTT

--models rf

--add_category stage

Build model

Use Rscript

Rscript regression.R --file all.args

Load tuned.Rdata and extract features sorted by importance score

Building Models:

Extract selected OTUs from otu_table. .prebiotics.even1007.txt (named M.txt)

1, physiological features

Create args file

--input_otu_table M.txt

--metadata meta.model.txt

--fields OGTT

--models rf

--cores 25

--add_category stage

Build model

Use Rscript

Rscript regression.R --file M.args

2, selected microbiome features

Create a control file with 1 feature with 0 abundance in every sample. (named control.txt)

Create args file

--input_otu_table control.txt

--metadata meta.model.txt

--fields OGTT

--models rf

--cores 25

--add_category stage

--add_numeric FPG,BMI,BF,BM,BM,SF,VF,initialOGTT

Build model

Use Rscript

Rscript regression.R --file physiology.args

3, selected microbiome & physiological features

Create args file

--input_otu_table M.txt

--metadata meta.model.txt

--fields OGTT

--models rf

--cores 25

--add_category stage

--add_numeric FPG,BMI,BF,BM,BM,SF,VF,initialOGTT

Build model

Use Rscript

Rscript regression.R --file M.physiology.args

Features Network

Convert otu_table.txt to otu_table.biom

biom convert -i M.txt -o M.biom --to-hdf5 --table-type="OTU table" --process-obs-metadata taxonomy

Generate taxa with abundance

summarize_taxa_through_plots.py -i M.bymodel.biom -o taxa.sankey -m meta.model.txt -c w1shout

Use perl script: create_cytoscape_input.pl to create a raw format input for Sankey plot

perl create_cytoscape_input.pl <taxonomy_dir> <output_dir> <otu_table.txt_prefix>

Fix the link and node file into Sankey plot format

Use R script: sankey.R to make Network plot

CONSORT 2010 checklist of information to include when reporting a randomised trial*

Section/Topic	Item No	Checklist item	Reported on page No
**Title and abstract**			
	1a	Identification as a randomised trial in the title	No reported
	1b	Structured summary of trial design, methods, results, and conclusions (for specific guidance see CONSORT for abstracts)	Page 1
**Introduction**			
Background and objectives	2a	Scientific background and explanation of rationale	Line 28- 44
	2b	Specific objectives or hypotheses	Line 45-52
**Methods**			
Trial design	3a	Description of trial design (such as parallel, factorial) including allocation ratio	Line248
	3b	Important changes to methods after trial commencement (such as eligibility criteria), with reasons	No change
Participants	4a	Eligibility criteria for participants	Line 228-232
	4b	Settings and locations where the data were collected	Line 220-221
Interventions	5	The interventions for each group with sufficient details to allow replication, including how and when they were actually administered	Line 234-247
Outcomes	6a	Completely defined pre-specified primary and secondary outcome measures, including how and when they were assessed	Line 251-260
	6b	Any changes to trial outcomes after the trial commenced, with reasons	No change
Sample size	7a	How sample size was determined	Line 228
	7b	When applicable, explanation of any interim analyses and stopping guidelines	No reported
Randomisation:			
Sequence generation	8a	Method used to generate the random allocation sequence	No reported
	8b	Type of randomisation; details of any restriction (such as blocking and block size)	Line 238
Allocation concealment mechanism	9	Mechanism used to implement the random allocation sequence (such as sequentially numbered containers), describing any steps taken to conceal the sequence until interventions were assigned	No reported
Implementation	10	Who generated the random allocation sequence, who enrolled participants, and who assigned participants to interventions	No reported
Blinding	11a	If done, who was blinded after assignment to interventions (for example, participants, care providers, those assessing outcomes) and how	Line 263-264
	11b	If relevant, description of the similarity of interventions	No reported
Statistical methods	12a	Statistical methods used to compare groups for primary and secondary outcomes	Line 307-311
	12b	Methods for additional analyses, such as subgroup analyses and adjusted analyses	Line 311-314
**Results**			
Participant flow (a	13a	For each group, the numbers of participants who were randomly assigned, received intended treatment, and were analysed	Line 55-56

diagram is strongly recommended)		for the primary outcome	
	13b	For each group, losses and exclusions after randomisation, together with reasons	Line 55-56
Recruitment	14a	Dates defining the periods of recruitment and follow-up	Line226-227
	14b	Why the trial ended or was stopped	No reported
Baseline data	15	A table showing baseline demographic and clinical characteristics for each group	Line 57
Numbers analysed	16	For each group, number of participants (denominator) included in each analysis and whether the analysis was by original assigned groups	Line55-56
Outcomes and estimation	17a	For each primary and secondary outcome, results for each group, and the estimated effect size and its precision (such as 95% confidence interval)	Line 63-70<br>Line 72-96
	17b	For binary outcomes, presentation of both absolute and relative effect sizes is recommended	No reported
Ancillary analyses	18	Results of any other analyses performed, including subgroup analyses and adjusted analyses, distinguishing pre-specified from exploratory	Line 99-141
Harms	19	All important harms or unintended effects in each group (for specific guidance see CONSORT for harms)	Line 58-61
**Discussion**			
Limitations	20	Trial limitations, addressing sources of potential bias, imprecision, and, if relevant, multiplicity of analyses	Line 213-217
Generalisability	21	General is ability (external validity, applicability) of the trial findings	Line 145-168
Interpretation	22	Interpretation consistent with results, balancing benefits and harms, and considering other relevant evidence	Line 178-195
**Other information**			
Registration	23	Registration number and name of trial registry	Line 220-224
Protocol	24	Where the full trial protocol can be accessed, if available	Line 248-249
Funding	25	Sources of funding and other support (such as supply of drugs), role of funders	Page 11
